# Reliability of Orthodontic Miniscrews: Bending and Maximum Load of Different Ti-6Al-4V Titanium and Stainless Steel Temporary Anchorage Devices (TADs)

**DOI:** 10.3390/ma11071138

**Published:** 2018-07-05

**Authors:** Andrea Scribante, Mona A. Montasser, Eman Saad Radwan, Luisa Bernardinelli, Roberto Alcozer, Paola Gandini, Maria Francesca Sfondrini

**Affiliations:** 1 Unit of Orthodontics and Paediatric Dentistry, Section of Dentistry, Department of Clinical, Surgical, Diagnostic and Paediatric Sciences, University of Pavia, 27100 Pavia, Italy; roberto.alcozer@gmail.com (R.A.); paola.gandini@unipv.it (P.G.); francesca.sfondrini@unipv.it (M.F.S.); 2 Orthodontic Department, Faculty of Dentistry, Mansoura University, Mansoura 35511, Egypt; mmontasser11@yahoo.com (M.A.M.); Dr.EmanSaad@hotmail.com (E.S.R.); 3 Section of Statistics, Department of Brain and Behavioural Sciences, University of Pavia, 27100 Pavia, Italy; luisa.bernardinelli@unipv.it

**Keywords:** anchorage, bend, dentistry, fracture, implant, load, miniscrew, orthodontics, shear

## Abstract

Temporary anchorage devices (TADs) have been introduced into orthodontic clinical practice in order to allow tooth movements while avoiding strain on adjacent teeth. Miniscrews are available in the market with different diameters and materials. Accordingly, the purpose of the present report was to measure and compare the forces to bend and fracture different mini implants. Ti-6Al-4V titanium and stainless steel TADs of different manufacturers (Spider ScrewHDC; Mini Implants–Leone; Benefit–Orteam; Storm–Kristal) were evaluated. Two different diameters (1.5 mm and 2.0 mm) were tested. The sample included 10 unused specimens for each group, blocked in an Instron Universal Testing Machine, and a shear load was applied at the neck of the miniscrew. The force to bend the miniscrew was measured at 0.1 mm and 0.2 mm deflections. Also, the maximum force before screw fracture was recorded. Data were submitted for statistical analysis. Results showed significantly higher forces for 2.0 mm than 1.5 mm screws, both at 0.1 mm and 0.2 mm deflections and at maximum load. Moreover, no significant differences were reported between titanium and stainless steel miniscrews of equal diameters.

## 1. Introduction

During orthodontic treatment, excessive forces have been associated with undesirable reactions and side effects, including bone hyalinization, root resorption, pain, patient discomfort, and anchorage loss [1]. Orthodontic miniscrews have been introduced as temporary anchorage devices (TADs): they allow skeletal anchorage for dental movements, thus decreasing the side effects of anchorage loss. Moreover, they permit the management of different orofacial deformities [2]. Several reports showed the use of miniscrews for space management [3], intrusion [4], extrusion [5], the retraction of anterior teeth [6], crossbite correction [7], and sliding mechanics [8]. The use of temporary anchorage devices (TADs) has been reported also for non-conventional purposes, as stabilization for facemask protraction [9], fracture management [10], palatal skeletal expanders [11], and provisional miniscrew-supported pontics [12].

Orthodontic miniscrews of different lengths (usually 5.0 mm to 10.0 mm) and diameters (ranging usually from 1.2 mm to 2.5 mm) are present in the market. Generally, larger diameter screws provide greater anchorage resistance than smaller diameters [13] and present lower fracture risk under torque loads [14]. On the other hand, miniscrews with a smaller diameter are more easily inserted in narrow spaces with lower risk of radicular damage [15]. The TADs that are most frequently employed in clinical practice usually present a diameter of 1.5 mm [16]. Generally, manufacturers supply titanium miniscrews, but stainless steel miniscrews are also present in the market. Stainless steel miniscrews had been tested for biocompatibility, showing no cytotoxic effects at low pH values [17]. Titanium biomedical devices also showed high biocompatibility with no cytotoxic effects [18]. Although bands, archwires, and auxiliary cell alterations of variable intensities may occur during orthodontic treatment with brackets, no cytotoxic effects have been reported; metals, nickel, and chromium release from orthodontic appliances has been demonstrated [19,20,21,22].

Osseointegration is not needed as for orthodontic miniscrews as it is for conventional dental implants, because mechanical retention is the determining factor for their primary stability [23]. However, the major clinical unwanted adverse event with TADs is the fracture of the miniscrew, which implies surgical removal of the broken part [24]. Orthodontic miniscrews have been tested extensively in vitro, and many mechanical parameters have been investigated in order to evaluate the factors that are related to increased fracture risk. Plastic deformation [25], insertion [26], and removal torque [27] have been evaluated.

The knowledge of initial bending and the maximum load of different miniscrews could help clinicians regarding choice of miniscrew material and diameter. Today, there are no studies that have evaluated the bending and fracture resistance of different miniscrews under tangential load. Moreover, there are no reports that evaluated stainless steel versus titanium miniscrews.

Therefore, the purpose of the present investigation was to measure and compare forces to bend (for 0.1 mm and 0.2 mm) and fracture both titanium and stainless steel miniscrews of two different diameters (1.5 mm and 2.0 mm) under shear load. The null hypothesis of the study was that there is no significant difference among the various groups tested.

## 2. Materials and Methods

**Figure 1:**
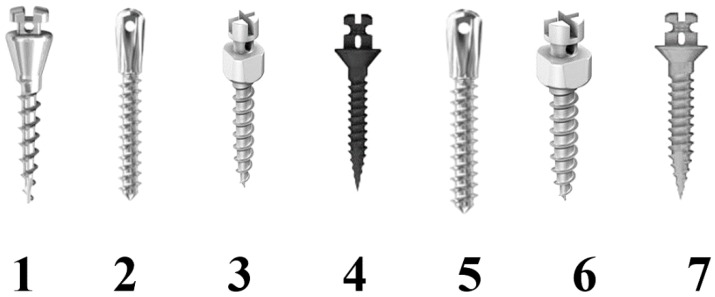
Miniscrews with different diameters tested in the present investigation. 1: 1.5 mm HDC—2: 1.5 mm Leone—3: 1.5 mm Orteam—4: 1.5 mm Kristal—5: 2.0 mm Leone—6: 2.0 mm Orteam—7: 2.0 mm Kristal.

In the present investigation, different Ti-6Al-4V titanium and stainless steel orthodontic miniscrews were evaluated (Figure 1).

Seven different screws were tested (Table 1): 1.5 mm (Spider Screw, HDC, Sarcedo, Italy); 1.5 mm (Mini implants, Leone, Sesto Fiorentino, Italy); 1.5 mm (Benefit, Orteam, Milano, Italy); 1.5 mm (Storm; Kristal, Trezzano Sul Naviglio, Italy); 2.0 mm (Mini implants, Leone, Sesto Fiorentino, Italy); 2.0 mm (Benefit, Orteam, Milano, Italy); 2.0 mm (Storm; Kristal, Trezzano Sul Naviglio, Italy).

**Figure 2:**
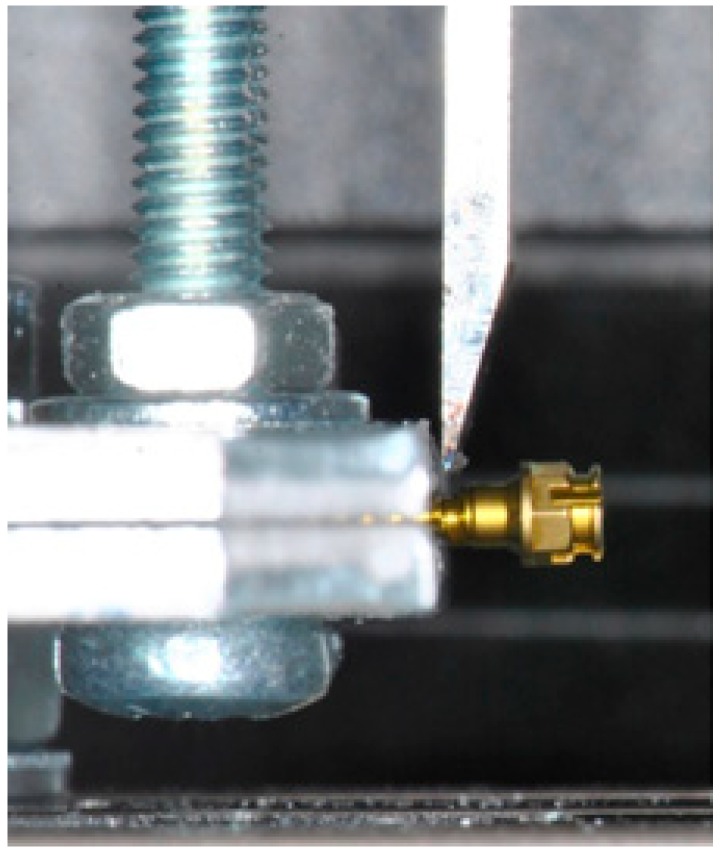
Photograph of the loading test set-up.

For each screw, 10 different new specimens were tested with a Universal Testing Machine (Instron, Norwood, MA, USA). Each mini implant was blocked in the lower jaw of the machine. The head (between the endoosseous thread and transmucosal collar) was exposed to tangential load (Figure 2) with a 1 mm/min crosshead speed [28,29].

Bending force was measured at 0.1 mm (groups 1 to 7) and 0.2 mm (groups 8 to 14) deflections. Moreover, the maximum load before screw fracture was recorded (groups 15 to 21). Load values were reported in Newtons [30,31].

Statistical analysis was performed with a computer software (R version 3.1.3, R Development Core Team, R Foundation for Statistical Computing, Wien, Austria). Mean, standard deviation, minimum, median, and maximum were chosen for descriptive statistics and were calculated for the 21 groups. The Kolmogorov–Smirnov test assessed Gaussian data distribution. ANOVA (analysis of variance) and Tukey tests were applied both for N and MPa data. Significance was predetermined at *p* < 0.05 for all statistical tests.

## 3. Results

Table 2 reports descriptive statistics of the force values (N) recorded in the 21 groups, including mean, standard deviation, minimum, median, and maximum.

**Figure 3:**
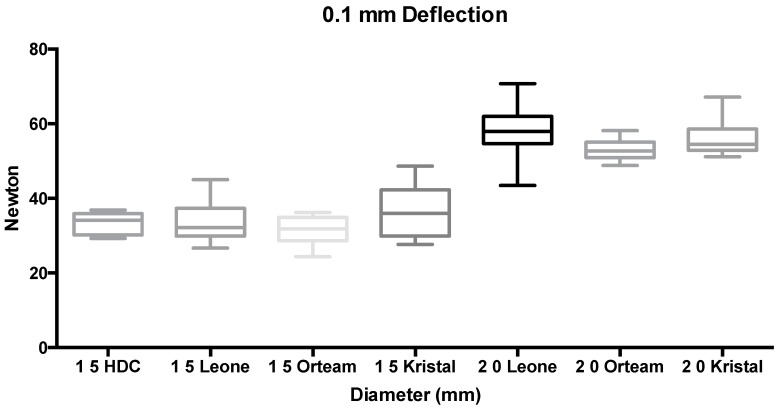
Box plot of groups tested at 0.1-mm deflection (N).

**Figure 4:**
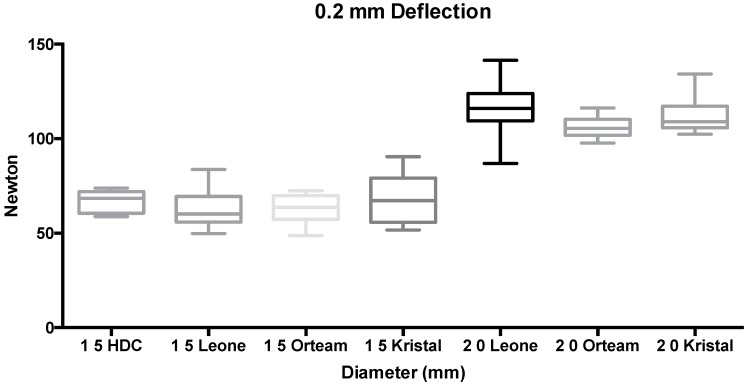
Box plot of groups tested at 0.2-mm deflection (N).

ANOVA showed the presence of significant differences among groups (*p* < 0.001). Tukey’s test used as a post hoc test reported that at both 0.1 mm and at 0.2 mm deflections, no significant differences were detected among the 1.5-mm diameter miniscrews (groups 1 to 4 and 8 to 11) (*p* > 0.05). Significantly higher forces (*p* < 0.05) were reported for 2.0-mm diameter TADs (groups 5 to 7 and 12 to 14), which showed no significant differences among them (*p* > 0.05) (Figure 3 and Figure 4).

**Figure 5:**
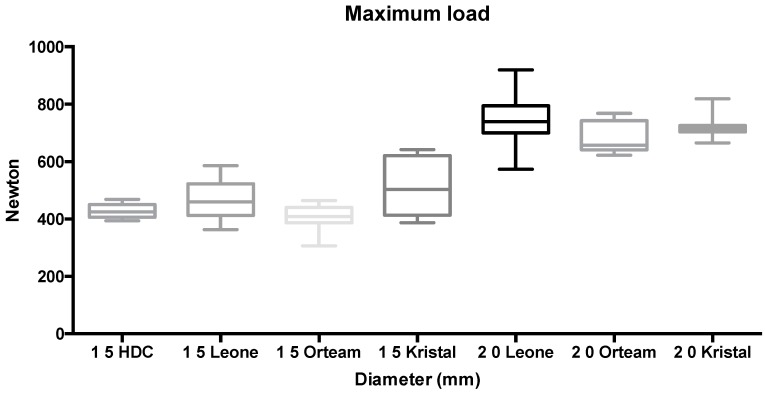
Box plot of groups tested at maximum load before fracture (N).

Similar results were reported at maximum load before screw fracture (Figure 5—groups 15 to 21).

No significant differences were reported between the titanium and stainless steel screws with the same diameter (*p* > 0.05).

**Figure 6:**
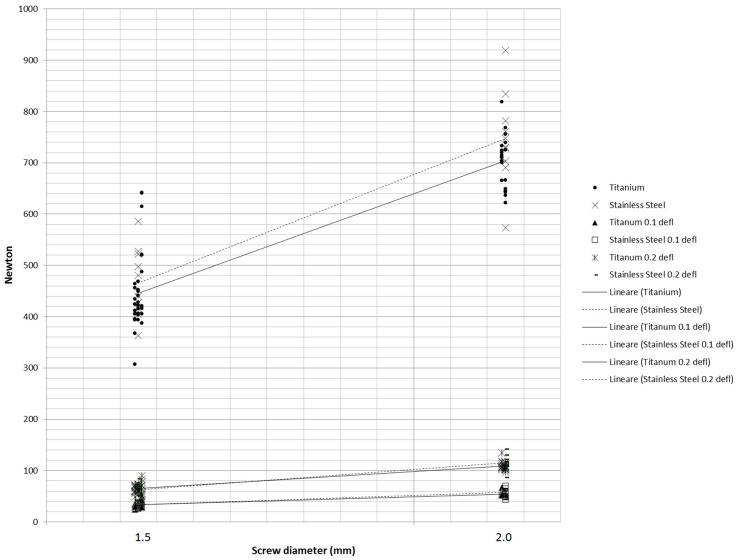
Linear regressions of fracture load values (N) plotted against to the diameter of the collar of the miniscrews in the three different testing conditions (0.1 mm deflection, 0.2 mm deflection, and maximum load before fracture).

Linear regressions (Figure 6) showed a significant effect of miniscrew diameter on force values recorded at 0.1 mm (*p* < 0.0001) and 0.2 mm (*p* < 0.0001) deflections and at maximum load (*p* < 0.001).

## 4. Discussion

Orthodontic miniscrews have been previously tested both in vitro [13,14,32,33] and in vivo [3,4,5,6,7,8]. Previous studies that investigated the clinical reliability of orthodontic miniscrews showed their effectiveness for skeletal anchorage in orthodontics, with a high success rate (about 80–90%) [34,35]. Previous authors have considered that, if miniscrew failure is the most frequent drawback during TADs employment, the screw fracture is the most unwanted complication [15]. In fact, a broken miniscrew has to be removed from bone, with an intervention that is neither easy nor always successful. For these reasons, sometimes broken miniscrews are left in the bone [15]. When miniscrews are used for orthodontic anchorage, the fracture risk is relatively low (about 1%) [36]. In fact, in the present study, the mean fracture values that were reported ranged between 405 N (1.5-mm diameter–titanium) and 747 N (2-mm diameter–stainless steel). The mean bending values at 0.1-mm deflection ranged between 31–58 N, whereas at 0.2-mm deflection, forces ranged between 43–116 N. All of these values are generously above the values reported during conventional clinical applications, which have been reported to be approximately under 5 N [37]. However, when miniscrews are used for non-conventional applications, fracture risk could increase, as miniscrews would be subjected to higher forces if compared with conventional orthodontic anchorage uses [10,11,12], even if no reports have evaluated this amount yet.

In the present report, miniscrews have been tested in air for tangential load as this vector of force is the same to which miniscrews are subjected when employed for unconventional orthopedic uses [16]. Moreover, during these applications, forces generated at screw collar are significantly higher than those generated when the screw is used for conventional orthodontic anchorage. Therefore, higher resistance to plastic deformation and fracture is needed [15]. The collar region can be considered the weak point of the whole screw, and this is the main reason for which the bending and fracture force have been applied at this specific point of the miniscrews in the present investigation.

The null hypothesis of the present study has been rejected; 2.0-mm diameter miniscrews showed significantly higher bending and fracture loads than 1.5-mm diameter miniscrews. However, no studies evaluated in air bending or fracture loads; therefore, the results of the present investigation are not directly comparable with the existing literature. On the other hand, many authors have studied insertion and removal torque loads, showing a significant effect of screw diameter. In fact, lower forces were recorded with small-diameter miniscrews, whereas higher values were found with larger diameter miniscrews [38]. This is in agreement with the present report, both when evaluating bending and fracture loads after shear force application.

Miniscrews are marketed with different lengths and diameters. During clinical practice, long screws (≥8 mm) present significantly higher success rates than the rates obtained with shorter ones (<8 mm) [33]. However, it has been demonstrated that as the length of the miniscrew that is in contact with the bone is increased, the amount and pattern of stress distribution in the cortical bone and the miniscrew do not change significantly [32]. For this reason, the miniscrew length has not been considered as a variable in the present investigation.

There are very few studies that have compared the performance and the mechanical properties of orthodontic miniscrews of different materials. Pan et al. found that despite their many differences, both titanium and stainless steel alloys meet the mechanical requirements for stable miniscrew implants [39]. Other studies showed no significant differences between the two materials—stainless steel and titanium—regarding histological responses with or without loading [40], and also regarding the percent of bone-to-implant contact or the static and dynamic bone parameters [41].

Although the modulus of elasticity of stainless steel (~193 GPa) and titanium Grade 5 (~120 GPa) are different, in the present study, no significant differences were recorded in the bending and fracture loads between the two materials, for both diameters tested (1.5 mm and 2.0 mm). However, Carano et al. [42] showed that stainless steel miniscrews started bending at lower level of forces, but failure at load was twice that of the titanium minscrews. They concluded that although stainless steel miniscrews demonstrated more resistant to failure, the overall performance of stainless steel as material for orthodontic miniscrews could be inferior to titanium. A strength point that could be added to the design of future studies would be testing miniscrews of the different materials manufactured with the same geometric design. Geometric design had been found to affect the mechanical properties of orthodontic miniscrews [43,44].

In our study, all of the screws that were evaluated were new. No tests have been conducted on retrieved mini implants. In dentistry, some materials are reused after disinfection and sterilization procedures. In vitro [45] and in vivo [46] studies demonstrated the reliability of many reconditioned orthodontic devices. In addition, miniscrews [47,48] have been tested after recycling, showing that morphological changes mainly occurred at the screw tip. The cortical bone penetration success rate of recycled screws has been reported to be lower than that of unused screws. On the other hand, no significant difference in the bone–miniscrew contact ratio has been found between new and used miniscrews [48]. Moreover, also fracture torque has been showed to be not influenced by recycling protocols [47]. However, future reports are needed in order to test the bending and fracture loads for retrieved miniscrews before suggesting clinical use.

Even if nowadays there is no evidence of differences in the mechanical properties of orthodontic miniscrews made of stainless steel or titanium alloys, of course, “the absence of evidence is not an evidence of absence”, and future studies on the topic are always welcomed [49].

## 5. Conclusions

Within the limitations of this in vitro study, the results demonstrated that:

1. Miniscrews of 2.0-mm diameter showed significantly higher bending and fracture resistance than 1.5-mm diameter TADs;
2. No significant differences were reported between titanium and stainless steel miniscrews with the same diameter;
3. Based on these results, when placing a miniscrew for non-conventional TADs’ applications, or when maximum bending and fracture resistance are needed, in order to reduce the risk of unwanted fracture due to tangential forces, a larger diameter is safer regardless of the miniscrew material.

## Figures and Tables

**Table 1:** Materials tested.

Name	Manufacturer	Diameter	Length	Material	*n*
Spider Screw	HDC	1.5 mm	10 mm	Titanium Ti-6Al-4V (Grade 5)	10
Mini Implants	Leone	1.5 mm	10 mm	Stainless Steel	10
Benefit	Orteam	1.5 mm	11 mm	Titanium Ti-6Al-4V (Grade 5)	10
Storm	Kristal	1.5 mm	10 mm	Titanium Ti-6Al-4V (Grade 5)	10
Mini Implants	Leone	2.0 mm	10 mm	Stainless Steel	10
Benefit	Orteam	2.0 mm	11 mm	Titanium Ti-6Al-4V (Grade 5)	10
Storm	Kristal	2.0 mm	10 mm	Titanium Ti-6Al-4V (Grade 5)	10

**Table 2:** Descriptive statistics of maximum force values (N) of the 21 groups tested (each group consisted of 10 specimens).

Group	Diameter	Deflection	Mean	SD	Min	Mdn	Max	Tukey *
1	1.5 mm	0.1 mm	33.28	2.92	29.28	34.16	36.86	A
2	1.5 mm	0.1 mm	34.69	9.50	21.13	33.38	53.70	A
3	1.5 mm	0.1 mm	31.53	3.86	24.34	31.81	36.21	A
4	1.5 mm	0.1 mm	36.38	7.01	27.67	36.00	48.65	A
5	2.0 mm	0.1 mm	58.00	7.17	43.45	57.99	70.73	B
6	2.0 mm	0.1 mm	53.07	2.92	48.84	52.71	58.16	B
7	2.0 mm	0.1 mm	55.21	8.58	38.58	53.63	69.32	B
8	1.5 mm	0.2 mm	66.57	5.85	58.56	68.32	73.73	B
9	1.5 mm	0.2 mm	64.71	17.71	39.48	62.29	100.42	B
10	1.5 mm	0.2 mm	63.05	7.72	48.68	63.62	72.42	B
11	1.5 mm	0.2 mm	67.87	13.06	51.66	67.19	90.44	B
12	2.0 mm	0.2 mm	116.00	14.34	86.91	115.98	141.46	C
13	2.0 mm	0.2 mm	106.15	5.84	97.68	105.41	116.31	C
14	2.0 mm	0.2 mm	110.43	17.16	77.16	107.26	138.64	C
15	1.5 mm	Maximum load	428.03	24.29	393.61	424.80	468.61	D
16	1.5 mm	Maximum load	481.60	133.40	300.08	462.41	773.25	D
17	1.5 mm	Maximum load	405.89	45.53	306.68	408.58	464.25	D
18	1.5 mm	Maximum load	505.67	99.29	387.43	503.65	642.12	D
19	2.0 mm	Maximum load	747.16	90.98	573.60	739.06	919.52	E
20	2.0 mm	Maximum load	685.03	55.53	622.47	657.79	768.21	E
21	2.0 mm	Maximum load	711.78	106.73	462.97	717.64	873.41	E

*: Mean with same letters are not significantly different.
